# Secondary Malignancy Risk Following Proton Radiation Therapy

**DOI:** 10.3389/fonc.2015.00261

**Published:** 2015-11-26

**Authors:** Bree R. Eaton, Shannon M. MacDonald, Torunn I. Yock, Nancy J. Tarbell

**Affiliations:** ^1^Department of Radiation Oncology, Massachusetts General Hospital, Harvard Medical School, Boston, MA, USA

**Keywords:** proton, radiotherapy, radiation, second malignancy

## Abstract

Radiation-induced secondary malignancies are a significant, yet uncommon cause of morbidity and mortality among cancer survivors. Secondary malignancy risk is dependent upon multiple factors including patient age, the biological and genetic predisposition of the individual, the volume and location of tissue irradiated, and the dose of radiation received. Proton therapy (PRT) is an advanced particle therapy with unique dosimetric properties resulting in reduced entrance dose and minimal to no exit dose when compared with standard photon radiation therapy. Multiple dosimetric studies in varying cancer subtypes have demonstrated that PRT enables the delivery of adequate target volume coverage with reduced integral dose delivered to surrounding tissues, and modeling studies taking into account dosimetry and radiation cell biology have estimated a significantly reduced risk of radiation-induced secondary malignancy with PRT. Clinical data are emerging supporting the lower incidence of secondary malignancies after PRT compared with historical photon data, though longer follow-up in proton treated cohorts is awaited. This article reviews the current dosimetric and clinical literature evaluating the incidence of and risk factors associated with radiation-induced secondary malignancy following PRT.

## Introduction

Radiation-induced secondary malignancies are a rare, yet significant late effect of radiation treatment among cancer survivors. The second malignancy risk is dependent upon the patient’s age, the radiation dose received and volume of normal tissue irradiated, as well as the patients’ family history of cancer and unique biological risk for malignancy (1, 2). As the risk is life-long and cumulative, children and young adults expected to survive many decades following definitive cancer therapy are at greatest risk for developing a radiation-induced malignancy. Long-term follow-up of the Childhood Cancer Survivor Study has demonstrated that there has been an increase in mortality attributed to second malignancies over time and the death rate due to a subsequent malignancy exceeds that due to all other causes at 25 years after first cancer diagnosis (3, 4). Retrospective series of large cohorts of pediatric patients treated with older photon radiotherapy techniques have reported a cumulative incidence of second malignancies ranging from 9.3 to 19% at 30 years (1, 5, 6), which can have profound effects on patient quality of life and mortality (4–6).

Proton therapy (PRT) is an advanced radiation technique now used with the hope of reducing late effects of radiotherapy. A proton beam has a unique dose-deposition pattern characterized by reduced entrance dose and minimal to no exit dose compared with conventional photon irradiation (7). Treating with protons gives the radiation oncologist the ability to maintain target volume coverage required for efficacious therapy, while minimizing dose delivered to nearby normal tissues (8). A primary expected benefit of this decrease in dose to normal tissues is reduced risk of secondary malignancies (9) as well as other radiation-induced acute and late effects. In this article, we will review selected dosimetric and clinic data addressing the impact of PRT treatment on secondary malignancy risk.

## Dosimetric Comparisons

**Figure 1:**
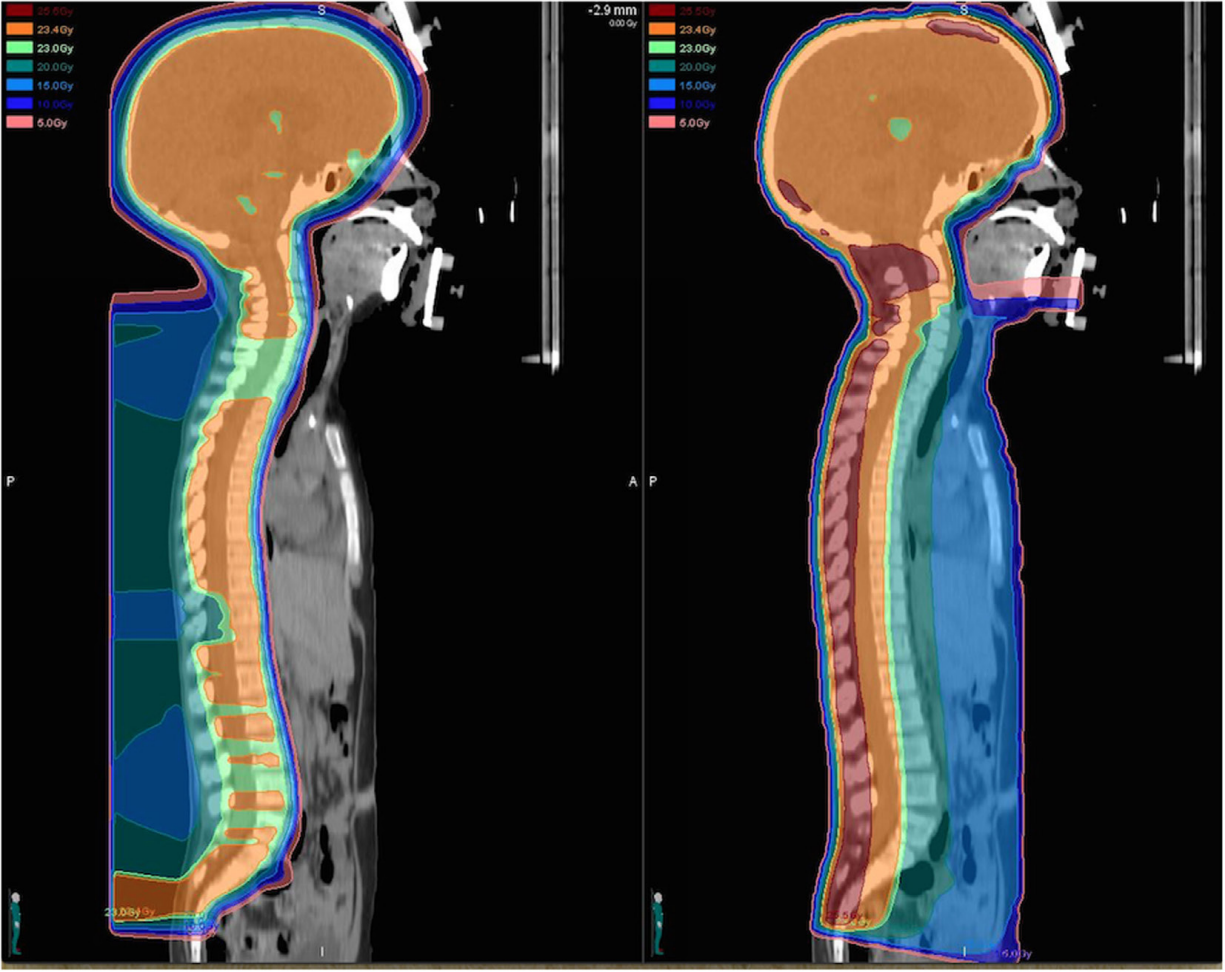
**Dose distributions for a proton (left) and photon (right) craniospinal plan prescribed to 23.4 Gy (relative biological equivalents) are illustrated for comparison**. The proton craniospinal plan provides considerable sparing of normal tissues anterior to the spinal canal and delivers a significantly reduced total integral dose to the patient.

In a treatment planning comparison study evaluating PRT in comparison to standard photon techniques and intensity-modulated radiation therapy (IMRT) for a variety of malignancies, the use of protons has been demonstrated to substantially reduce the volume of normal tissues receiving medium to low doses (below about 70% of the target dose) when compared with both standard and IMRT photon plans (8). Over all cases, the use of protons lead to a reduction of the total integral dose by a factor of three compared to standard photon techniques and at least a factor of two compared to intensity-modulated photon plans (8). Many similar dosimetric comparison studies have been performed for a variety of specific tumor types among children and adults and have clearly demonstrated the superior ability of PRT to spare normal tissues from unwanted radiation (9–12). With the use of craniospinal irradiation for medulloblastoma, the dosimetric benefit of protons is particularly striking as organs anterior to the vertebrae are spared from receiving unwanted radiation with PRT (12) (Figure 1). In effort to quantify the effect of the reduced total integral dose delivered with PRT on secondary malignancy risk, studies have further utilized modeling systems based on dosimetric comparisons, organ equivalent dose, and radiation protection models to approximate the benefit of protons with regard to second malignancies.

In an analysis of pediatric rhabdomyosarcoma and medulloblastoma cases, Miralbell et al. (13) analyzed the dosimetry of conventional photons, IMRT, and scattered and scanned proton plans and estimated secondary cancer risk according to the International Commission on Radiologic Protection (ICRP). Results revealed that proton plans reduced the expected incidence of radiation-induced secondary cancers for the rhabdomyosarcoma case by a factor ≥2 and for the medulloblastoma case by a factor of 8–15 when compared with either IMRT or conventional X-ray plans (13). Furthermore, cost-effectiveness analysis which has included the risk of secondary malignancies for patients with medulloblastoma has shown that proton treatment is associated with higher quality adjusted life years and reduced cost, in part due to the estimations of reduced incidence of secondary malignancies (14, 15). In a similar study from Moteabbed et al. (16), dose distributions from passive scattered protons, pencil bean scanning protons, and IMRT and volumetric-modulated arc therapy (VMAT) photon plans for six pediatric patients with brain and head-and-neck tumors were used to calculate the excess absolute risk (EAR) and lifetime attributable risk (LAR) for developing a second tumor in the soft tissue and skull. The LAR for IMRT/VMAT relative to proton plans ranged from 1.3 to 4.6 for soft tissue and from 3.5 to 9.5 for the skull. Larger absolute LAR was observed for younger patients and when using linear risk models (16). Paganetti et al. (17) used phantom data and a sophisticated risk model based on cell kill, mutation, repopulation, and inhomogeneous organ doses to estimate the LAR of second malignancy within the RT field for representative cases of optic glioma and vertebral body Ewing’s sarcoma on a 4- and 14-year-old fully contoured phantom. This study found that protons may reduce the risk of second malignancy by a factor ranging from 2 to 10 and also demonstrated that LAR was affected by different methods of proton RT planning (17).

Multiple other dosimetric and secondary risk modeling studies have been performed for adult malignancies with similar results. In a dosimetric comparison between PRT and IMRT among 11 patients with low-grade glioma prescribed 54 Gy (RBE), the equivalent uniform dose delivered to adjacent normal tissues was found to be 10–20 Gy lower with protons (18). Using biological modeling of radiation induced toxicities, the mean ratio for excess risk of radiation induced second tumors with IMRT as compared to protons was found to be 2.2 (range 1.6–6.5). The mean excess risk of a PRT induced second tumor in the brain per 10,000 cases per year was 47 (range 11–83), while the mean risk for IMRT was 106 (range 70–134) (18). In an analysis of conventional parallel opposed and intensity-modulated photon and proton treatment plans for a patient with Hodgkin’s disease, spot scanning PRT was found to decrease the secondary malignancy risk by a factor of 2 based on the ICRP calculation scheme and normal tissue dose distribution (19).

Yoon et al. (20) used ion chambers and CR-39 detectors to measure the secondary dose delivered to tissues outside of the target volume (measured at 20–60 cm from the isocenter) during irradiation with IMRT and PRT for patients with prostate and head-and-neck cancer and estimated organ-specific radiation-induced cancer risk by applying organ equivalent dose estimations to dose distributions. The average secondary doses for prostate patients ranged between 3 and 1 mSv/Gy with IMRT, which was approximately one order of magnitude higher than for PRT. Although the average secondary doses of IMRT were higher than those of PRT for head-and-neck cancers, these differences were not significant. Organ equivalent dose calculations showed that, for prostate cancer patients, the risk of secondary cancers in out-of-field organs, such as the stomach, lungs, and thyroid, was at least five times higher for IMRT than for PRT (20). A second dosimetric comparison among prostate cancer patients evaluating protons and 6 MV IMRT photon plans was performed by Fontenot et al. (21), and the secondary malignancy risk was estimated by taking into account both primary and secondary contributions to total dose delivered on an organ-specific basis and using risk models from the Committee on the Biological Effects of Ionizing Radiation. It was found that PRT reduced the risk of a secondary malignancy by 26–39% compared with IMRT, which was attributed to the substantial sparing of the rectum and bladder by the PRT plans in this study (21).

## Neutron Scatter

Secondary dose from neutron scatter produced during radiotherapy contributes to the total effective dose delivered to the patient and the secondary malignancy risk but is not accounted for in most dosimetric comparisons. The neutron scatter with PRT results from protons losing energy as they interact with the range modulator and apertures (22). In some circumstances, neutron dose delivered outside of target tissues with passively scattered protons has been estimated to be much greater than with either scanned PRT, intensity modulated, or conventional photon treatment (23). This has sparked controversy about the effect of PRT on secondary malignancy risk and the relative benefit of pencil beam scanning vs. passive scattering proton treatment (22–26).

The neutron dose equivalent with pencil beam scanning for a medium size target volume can reach approximately 1% of the treatment dose in the region of the Bragg peak (27). In non-target tissues of the patient, the neutron dose contributes approximately 0.004 and 0.002 Sv/treatment Gy, for large and medium target volumes, respectively, a factor which is about two times that of photon therapy (26). However, because this dose delivered to the non-target area from neutron scatter is far less than that resulting from primary dose fall-off, multiple authors have concluded that primary dosimetric comparisons are sufficient for estimation of secondary malignancy risk. Thus, the total integral dose delivered to the patient remains much less with either passive scattered or pencil bean scanning protons in comparison with photon treatment (24, 26).

Dosimetric comparisons taking into account the contribution of neutron scatter have concluded that PRT still delivers a reduced total integral dose when compared with photon irradiation (24). In an analysis of 30 patients with prostate cancer from Schneider et al. (27), the impact of X-ray scatter, neutron radiation, and the primary dose distribution on secondary cancer incidence were analyzed for convention and intensity-modulated photon plans as well as spot scanning PRT. After considering both primary dose and scatter dose contributions, it was estimated that the use of spot-scanned protons reduced the secondary cancer incidence by as much as 50% when compared with photon therapy (27). Pencil beam scanning PRT may provide the greatest opportunity to reduce the impact of neutron scatter on secondary malignancy risk as the out-of-field neutron dose produced by a scattered proton beam has been estimated to be more than 100 times that off a scanned proton beam (28). However, secondary malignancy risk due to scatter radiation from passively scattered proton beam treatment remains low. A study evaluating the total lifetime risk of a second cancer from stray radiation alone to be 1.5% for passively scatter craniospinal proton treatment and 0.8% for scanned craniospinal proton treatment (29). And when taking into account the therapeutic radiation as well as scatter radiation dose, passively scattered and scanned proton beam treatment similarly reduced the secondary cancer risk in comparison with IMRT photon treatment (29).

## Clinical Data

Clinical data comparing the secondary malignancy incidence in long-term survivors following proton and photon therapy are limited given the significant follow-up time necessary to effectively evaluate radiation induced malignancy. However, a reduced incidence of radiation induced second cancer with PRT as compared to photon therapy has been reported in multiple series with early follow-up. Chung et al. (30) performed a large case matched comparison of 588 patients treated with PRT at the Harvard cyclotron from 1973 to 2001 and 588 patients treated with photon therapy from the Surveillance, Epidemiology, and End Results Program. Patients were matched with respect to age at radiation treatment, sex, year of treatment, cancer histology, and treatment site, and the median follow-up time was 6.7 years for proton patients and 6.0 years for photon patients. The majority of patients were adults with tumors of the prostate, central nervous system or head-and-neck region. The crude rate of second malignancies was 5.2% among the proton cohort (29 patients) vs. 7.5% in photon cohort (42 patients). On multivariable analysis, PRT was associated with a decreased risk of second malignancy [adjusted hazard ratio, 0.52 (95% confidence interval, 0.32–0.85), *p* = 0.009] when compared with photon therapy (30).

Multiple clinical series of pediatric patients have reported excellent outcomes with very low rates of second malignancies after PRT (Table 1). Among a prospective phase II study of PRT for 59 children with medulloblastoma and a median follow-up of 7 years (range 3.9–10.3), no patients have suffered from a second malignancies (31). The results from the 45 standard risk patients from this phase II study were then compared to a case matched series of 43 patients treated with photons over a similar time period (32). Three patients from the photon cohort experienced a second malignancy, including an astrocytoma, intracranial desmoid tumor, and thyroid cancer occurring 12.9, 3.7, and 12.7 years after treatment, respectively, while none of the proton patients developed a second tumor (32). Sethi et al. (33) retrospectively analyzed the incidence of secondary malignancy among patients treated with either proton or photon therapy for retinoblastoma. After a median follow-up of 6.9 years (range, 1.0–24.4 years) for the 55 patients in the proton cohort and 13.1 years (range, 1.4–23.9 years) for the 31 patients in the photon cohort, the 10-year cumulative incidence of RT-induced or in-field second malignancies was significantly less among the proton cohort (0 vs. 14%, *p* = 0.015) (33). One proton patient with bilateral retinoblastoma did develop an out-of-field osteosarcoma of the femur (33). Other proton series among children with ependymoma (34), low-grade glioma (35), Ewing’s sarcoma (36), and rhabdomyosarcoma (37) have also reported no cases of PRT associated solid tumors (Table 1) after limited follow-up. Though longer follow-up is required for effective secondary malignancy risk comparison between proton and photon radiotherapy, these early clinical reports suggest a reduced incidence of radiation induced secondary cancers with PRT.

**Table 1:** **Secondary malignancy outcome data in pediatric patients treated with proton radiotherapy**.

Reference	Diagnosis	*N*	Follow-up median (range)	Secondary solid tumor incidence (%)
Yock et al. (31)	Medulloblastoma	59	7 years (3.9–10.3)	0
Greenberger et al. (35)	Low-grade glioma	32	7.6 years (3.2–18.2)	0
Sethi et al. (33)	Retinoblastoma	55	6.9 years (1.0–24.4)	5^a^
MacDonald et al. (34)	Ependymoma	70	3.8 years (1–11.7)	0
Ladra et al. (37)	Rhabdomyosarcoma	57	3.9 years (1.2–8.5)	0
Rombi et al. (36)	Ewings sarcoma	30	3.2 years (1.5–7.4)	0^b^

*^a^One patient with bilateral retinoblastoma developed a non-metastatic osteosarcoma of the femur*.

*^b^Four patients developed secondary hematologic malignancies*.

## Conclusion

Multiple dosimetric studies have demonstrated the ability of PRT to deliver efficacious target volume coverage while reducing the total integral dose delivered to normal tissues when compared with photon therapy. Modeling systems taking into account these dosimetric comparisons, organ equivalent dose and radiation protection models have predicted a significantly reduced risk of secondary malignancy with PRT among multiple pediatric and adult malignancies. Though neutron scatter may be higher in tissues outside of the target volume with proton treatment, the secondary dose contribution is small and thus the total integral dose remains less with protons than with photon therapy. Pencil beam scanning systems provide the greatest opportunity to reduced secondary dose from neutron scatter and further reduced the secondary malignancy risk from proton treatment. Though clinical data are limited, early reports of prospective and retrospective series suggest a reduced incidence of secondary malignancy in patients treated with protons, and further analyses with longer follow-up are awaited. The data support the continued use of PRT in effort to reduce the incidence of secondary malignancies among children and adults expected to survive their disease.

## Author Contributions

All authors contributed to the interpretation of the original research reviewed here, drafting the work or revising it critically for important intellectual content, and gave final approval of the version to be published. All authors agree to be accountable for all aspects of the work in ensuring that questions related to the accuracy or integrity of any part of the work are appropriately investigated and resolved.

## Conflict of Interest Statement

The authors declare that the research was conducted in the absence of any commercial or financial relationships that could be construed as a potential conflict of interest.
